# SHARE – workpackage 5 : evidence based recommendations for diagnosis and treatment of childhood-onset systemic lupus erythematosus

**DOI:** 10.1186/1546-0096-12-S1-P109

**Published:** 2014-09-17

**Authors:** Noortje Groot, Nienke de Graeff, Tadej Avcin, Brigitte Bader-Meunier, Paul Brogan, Pavla Dolezalova, Brian Feldman, Isabelle Kone-Paut, Pekka Lahdenne, Alberto Martini, Liza McCann, Seza Ozen, Clarissa Pilkington, Angelo Ravelli, Annet van Royen, Bas Vastert, Nico Wulffraat, Sylvia Kamphuis, Michael Beresford

**Affiliations:** 1 Alder Hey Children's Hospital, Liverpool, UK; 2 Erasmus MC-Sophia, Rotterdam, Netherlands; 3 University Medical Centre Utrecht, Utrecht, Netherlands; 4 University Medical Centre, Ljubljana, Slovenia; 5 Necker Hospital, Paris, France; 6 Great Ormond Street Hospital, London, UK; 7 General University Hospital, Prague, Czech Republic; 8 Sick Kids Hospital, Toronto, Canada; 9 CHU de Bigetre, Paris, France; 10 Children's Hospital of Helsinki and Uusimaa, Helsinki, Finland; 11 Gaslini Children's Hospital, Genova, Italy; 12 Hacettepe University Children's Hospital, Ankara, Turkey

## Introduction

Childhood-onset systemic lupus erythematosus (cSLE) is a rare multisystem autoimmune disease that often leads to significant morbidity. Evidence-based guidelines are sparse and management is mostly based on physician’s experience. Consequently, treatment regimens differ throughout Europe. In 2012, a European initiative called SHARE (Single Hub and Access point for paediatric Rheumatology in Europe) was launched to optimize and disseminate diagnostic and management regimens in Europe for children and young adults with rheumatic diseases such as cSLE.

## Objectives

To develop evidence-based recommendations for diagnosis and treatment of cSLE.

## Methods

Evidence based recommendations were developed using the European League Against Rheumatism (EULAR) standard operating procedure. An expert committee, consisting of paediatric rheumatologists from across Europe with expertise in cSLE, defined search terms for the systematic literature review. Two independent experts scored articles for validity and level of evidence. Through an online survey, experts evaluated recommendations derived from the literature. The recommendations were discussed at a consensus meeting using the nominal group technique^1^. Recommendations were accepted if more than 80% agreement was reached.

## Results

The literature search yielded 9341 articles, of which 128 (75 for diagnosis; 53 for treatment) were considered relevant and therefore scored for validity and level of evidence. Only sixteen articles (4 for diagnosis; 12 for treatment) were deemed valid; a larger number of articles were scored moderately valid (62 for diagnosis, 25 for treatment). Both were used in the formulation of the recommendations. Eighteen recommendations for diagnosis and 24 for treatment were suggested in the online survey. Thirteen recommendations for diagnosis and 14 for treatment were accepted with more than 80% agreement during the consensus meeting. Table 1 summarizes the categories of recommendations.

**Table 1:** 

Recommendations regarding:	Number
Diagnosis:	
Classification	3
Laboratory features	2
Access to other specialities	2
Comorbidities (growth/MAS/ heri-ditary conditions)	3
NP-SLE	
Acknowledgement of necessary research	2
	1

Treatment	
General	2
Haematological involvement	1
Lupus Nephritis (Class III/IV/V)	8
Compliance	1
NP-SLE	2

## Conclusion

The SHARE initiative provides recommendations for diagnosis and treatment for cSLE and thereby facilitates improvement and uniformity of care throughout Europe. Currently, a similar process is going on to add further guidelines including those on treatment and holistic care for PRD patients. As a final result, SHARE will provide standards of minimal care for different PRDs, including cSLE.

## Disclosure of interest

None declared.

